# Inflammation in sputum relates to progression of disease in subjects with COPD: a prospective descriptive study

**DOI:** 10.1186/1465-9921-7-136

**Published:** 2006-11-18

**Authors:** David G Parr, Andrew J White, Darren L Bayley, Peter J Guest, Robert A Stockley

**Affiliations:** 1 Department of Respiratory Medicine, University Hospitals of Coventry and Warwickshire, Clifford Bridge Road, Coventry, CV2 2DX, UK; 2 Department of Respiratory Medicine, Gloucester Royal Infirmary, Gloucester, UK; 3 Lung Investigation Unit, University Hospital of Birmingham, Edgebaston, Birmingham, B15 2TH, UK; 4 Department of Radiology, Queen Elizabeth Hospital, Edgebaston, Birmingham, B15 2TH, UK

## Abstract

**Background:**

Inflammation is considered to be of primary pathogenic importance in COPD but the evidence on which current understanding is based does not distinguish between cause and effect, and no single mechanism can account for the complex pathology. We performed a prospective longitudinal study of subjects with COPD that related markers of sputum inflammation at baseline to subsequent disease progression.

**Methods:**

A cohort of 56 patients with chronic bronchitis was characterized in the stable state at baseline and after an interval of four years, using physiological measures and CT densitometry. Sputum markers of airway inflammation were quantified at baseline from spontaneously produced sputum in a sub-group (n = 38), and inflammation severity was related to subsequent disease progression.

**Results:**

Physiological and CT measures indicated disease progression in the whole group. In the sub-group, sputum myeloperoxidase correlated with decline in FEV_1 _(rs = -0.344, p = 0.019, n = 37). LTB4 and albumin leakage correlated with TLCO decline (rs = -0.310, p = 0.033, rs = -0.401, p = 0.008, respectively, n = 35) and IL-8 correlated with progression of lung densitometric indices (rs = -0.464, p = 0.005, n = 38).

**Conclusion:**

The data support a principal causative role for neutrophilic inflammation in the pathogenesis of COPD and suggest that the measurement of sputum inflammatory markers may have a predictive role in clinical practice.

## Background

Chronic obstructive pulmonary disease (COPD) is a slowly progressive condition characterised by airflow obstruction that is 'not fully reversible' [1]. The main aetiological factor is cigarette smoking and it is generally accepted that the pathogenic mechanism is the development of an abnormal inflammatory response to inhaled particles and gases in the lung. Exposure to tobacco smoke appears to be universally associated with lung inflammation, but only a proportion of smokers develop COPD. Nevertheless, an enhanced inflammatory response has been demonstrated in the lungs of susceptible individuals that is more marked with continuing exposure to tobacco smoke [2] and the level of inflammation has been shown to relate to disease severity [3-6]. In addition, sputum neutrophilia has been related to FEV_1 _decline over the preceding 15 years [7]. Consequently, lung inflammation is likely to play a primary pathogenic role and offers a common unifying theme to the varied clinical phenotype seen in COPD [8]. However, the design of these studies does not allow cause to be distinguished from effect and, consequently, prospective studies that relate inflammation to disease progression are required to validate the inflammatory hypothesis of COPD causation. In addition, a prospective study design may also assist in resolving the lack of consensus over the relative importance of different inflammatory mechanisms.

Disease progression in longitudinal studies of COPD has traditionally been assessed using FEV_1 _but this is a non-specific measure of airflow obstruction that does not identify the relative contribution of the component parts of this heterogeneous syndrome. Co-existing impairment of gas exchange implies the presence of emphysema but this too is a non-specific measure and, in addition, emphysema is defined in morphological rather than physiological terms [9]. Consequently, the development of CT densitometry as a valid *in vivo *measure of emphysema [10,11] offers an opportunity to characterise individual phenotype in more detail and to clarify the contribution of emphysematous destruction to the decline in FEV_1_. In studies of subjects with alpha 1-antitrypsin deficiency (AATD), it has been shown to be a more sensitive measure of disease progression than either physiology or health status indices [12] and to relate to the decline in the gold standard method, namely FEV_1 _[13]. However, the clinical phenotype in usual COPD is generally considered to be more heterogeneous than in AATD and the role of CT densitometry in longitudinal studies is less well established.

The current study was performed to characterise, in detail, disease progression over a 4-year interval in a cohort of subjects with COPD and chronic bronchitis. Markers of neutrophilic inflammation in sputum were measured in a sub-group of individuals at baseline and were related to subsequent disease progression, assessed using traditional physiological measurements and contemporary CT densitometry.

## Methods

### Subjects

Subjects aged 40–80 years with smoking related chronic bronchitis [14] were recruited from two sources; firstly, from a cohort of subjects enrolled in a study of exacerbations of COPD (n = 46) [15] and secondly, from subjects attending a specialist out-patient COPD clinic (n = 10). Serum alpha 1-antitrypsin levels were assayed using immunoassay.

### Study design

Assessment at baseline included lung function testing, computed tomography with lung densitometry, characterisation of sputum inflammation and microbiology. Patients were invited to attend four years after their initial visit in order to assess disease progression using physiological and radiological criteria and, in addition, from changes in disease stage using the updated GOLD classification [16]. The study was approved by the local ethics committee and patients provided written informed consent.

### Sputum analysis

Spontaneously produced sputum samples were obtained at baseline in the stable clinical state, at least two months after any exacerbation. Samples were collected over four hours after waking and quantitative bacterial culture was performed on an aliquot [17]. The remaining material was centrifuged to obtain the sol-phase for the assay of myeloperoxidase (MPO), interleukin-8 (IL-8), leukotriene B4 (LTB4) and albumin as described previously [18]. Myeloperoxidase activity in sol-phase sputum was measured by chromogenic substrate assay relative to a known standard (Sigma Aldrich, Poole, Dorset, UK). IL-8 and LTB4 in sol-phase sputum were measured by ELISA using commercially available kits (Quantikine, R&D Systems Europe Ltd, Abingdon, UK and Amersham International plc, Buckinghamshire, UK, respectively). Sol-phase albumin was measured by radial immunodiffusion using a commercially available kit (The Binding Site Limited, Birmingham, UK). Ten ml of clotted venous blood was used to determine the serum albumin concentration using the same method given for sputum analysis, and the sputum/serum albumin ratio was calculated in order to assess airspace protein leakage.

### Lung function testing

Lung function testing was performed at baseline and 4 years later. Spirometry was performed using a wedge-bellows (Vitalograph Ltd, Buckinghamshire, UK) both before and following inhalation of 400 mcg of salbutamol and 60 mcg of ipratropium bromide via a large volume spacer device. Lung volumes were measured by helium dilution (Morgan Medical, Kent, UK), and diffusing capacity for carbon monoxide (Tlco) and transfer coefficient (KCO) was determined using the single breath method [19]. Baseline and follow-up measurements were performed on the same equipment according to national quality control guidelines [20], and results were expressed as a percentage of predicted values. Post-bronchodilation values were used for the assessment of decline in lung function.

### Computed tomography and lung densitometry

Whole lung computed tomography scans were performed at full inspiration, on a General Electric Prospeed scanner at baseline and four years later, using a high-resolution protocol (120kVp, 200mAs, 1 mm collimation at 10 mm intervals, 'bone' reconstruction algorithm). All scans were visually assessed for the presence of emphysema and bronchiectasis by a radiologist (P.J.G.) using established criteria [21,22].

Lung densitometry was performed on selected images at the level of the aortic arch ('Upper Zones') and the inferior pulmonary veins ('Lower Zones') using semi-automated computer software (Pulmo-CMS, MEDIS Medical Imaging Systems BV, Leiden, the Netherlands) as described previously [23]. Two densitometric indices were measured; the voxel index -950 (V.I.-950), which is defined as the proportion of lung voxels below a threshold of -950 Hounsfield Units (HU) and the 15^th ^percentile point (Perc15), which is the cut off value in HU below which 15% of voxels are distributed. These methods have been validated against pathology [10,11] and used in previous clinical studies [12,23,24].

A standardised rate of change was calculated for each parameter by dividing the difference between the two values by the time elapsed between the tests in order to overcome minor variability in the duration of follow-up.

### Statistical analysis

Statistical analyses were performed using the SPSS statistical programme (version 11.5 Chicago, Il). Data were tested for normality using the Shapiro-Wilk test. Normally distributed data are reported as the mean and standard deviation (SD) and comparisons made with the *t*-test. Non-parametric data are reported as the median and interquartile range [IQR]) and comparisons were made with the Wilcoxon signed rank test for paired data and the Mann Whitney-U test for grouped data. Relationships between variables were examined using Spearman's rank correlation test and categorical data were compared with the Chi-squared test. A *p *value of less than 0.05 was considered to be statistically significant.

## Results

### Baseline subject characteristics

**Figure 1:**
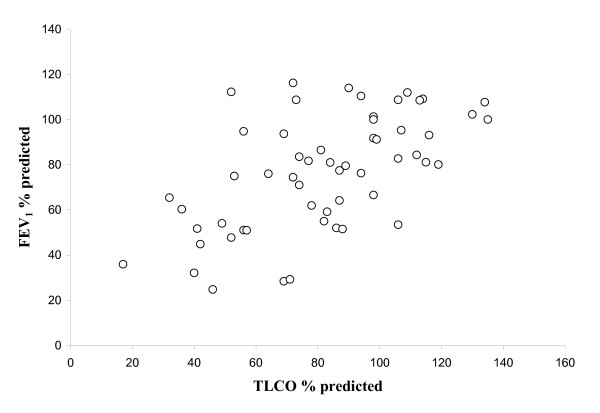
Individual data for the FEV1 and TLCO measurements at baseline, expressed as percent predicted, (n = 56).

**Figure 2:**
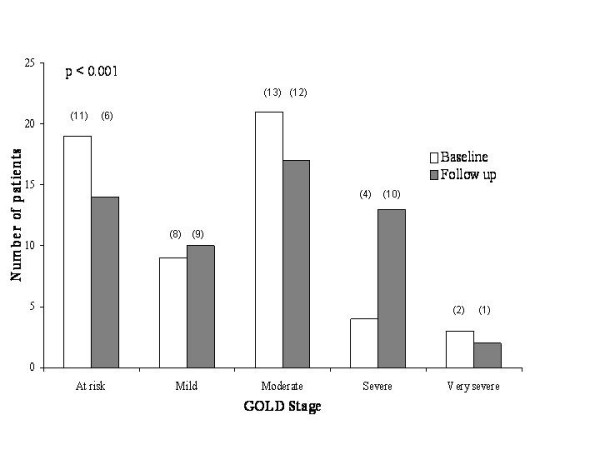
**Distribution of patients according to GOLD staging category at baseline (open histograms) and at follow-up, four years later (grey histograms)**. There was a significant redistribution in the disease stage towards more severe disease over the study period. The significance (p) is shown for the Wilcoxon signed rank test. The figures in brackets indicate the number of subjects within each group that contributed to data relating to sputum inflammation.

Sixty-five patients agreed to participate in the follow-up study but complete physiological and imaging data could only be obtained in 56 patients. All subjects had a history of chronic bronchitis [14] and no patients had alpha 1-antitrypsin deficiency. The mean (standard deviation – s.d.) age at baseline was 63 (7.4), and 29 (52%) of patients were male. More than half (30) of patients were current smokers and the mean (s.d.) number of pack years was 55 (39.9). The individual data for FEV_1 _(% predicted) and TLCO (% predicted) are indicated in Figure 1. The mean (s.d.) FEV_1 _was 1.89 L (0.71 L) (76.8 % predicted) and the mean (s.d.) FEV_1_/FVC was 56.6 (17.2). The baseline characteristics of the 38 patients with inflammatory data were comparable to the whole group (data not shown). The number of patients assigned to each GOLD category is indicated in Figure 2 and the baseline physiology for each category is shown in Table 1.

**Table 1:** Baseline characteristics of patients according to GOLD category

	**Stage 0 n = 19**	**Stage 1 n = 9**	**Stage 2 n = 21**	**Stage 3 n = 4**	**Stage 4 n = 3**
FEV_1 _(L)	2.54 (0.49)	2.06 (0.41)	1.57 (0.43)	0.96 (0.17)	0.73 (0.13)
FEV_1 _(%pred)	101.6 (10.1)	89.1 (11.2)	63.2 (10.3)	40.2 (7.4)	27.5 (2.3)
FVC (L)	3.39 (0.69)	3.61 (0.70)	3.38 (1.12)	3.17 (1.07)	2.83 (0.49)
FVC (%pred)	108.8 (9.6)	127.1 (22.2)	102.3 (20.4)	104.5 (14.0)	81.9 (16.4)
FEV_1_/FVC (%)	75.4 (6.0)	57.2 (4.5)	48.3 (10.0)	32.6 (9.5)	26.7 (7.6)
RV (L)	1.93 (0.41)	2.69 (0.56)	3.40 (0.92)	3.46 (1.02)	4.63 (1.06)
RV (%pred)	92.3 (12.7)	130.3 (24.6)	156.2 (36.5)	171.5 (34.2)	194.0 (40.5)
TLC (L)	5.44 (1.04)	6.25 (0.99)	6.59 (0.99)	6.41 (2.05)	7.01 (1.38)
TLC (%pred)	98.9 (7.9)	118.4 (12.9)	117.0 (19.3)	119.5 (17.6)	113.7 (20.5)
TLCO (mmol/min/kPa)	8.03 (2.10)	7.00 (2.28)	5.71 (2.09)	2.92 (1.18)	5.06 (1.43)
TLCO (%pred)	100.5 (21.2)	91.2 (24.3)	72.0 (21.3)	37.8 (14.8)	62.0 (13.9)
KCO (mmol/min/kPa/L)	1.60 (0.32)	1.33 (0.38)	1.14 (0.41)	0.69 (0.33)	1.14 (0.36)
KCO (%pred)	110.3 (23.4)	93.4 (31.6)	81.4 (29.2)	46.0 (20.9)	86.7 (27.5)

Figures represent the mean (standard deviation).

Spontaneous sputum was obtained from 47 patients at baseline. Microbiological assessment was performed on all samples but sufficient sputum sol-phase for inflammatory analysis was available from only 38 subjects. The baseline concentrations of markers of sputum inflammation are as follows, expressed as median (interquartile range), [number of patients]: myeloperoxidase, 0.33 units/ml (0.20–0.86) [n = 37]; leukotriene B4, 4.39nM (1.71–6.98) [n = 36]; interleukin 8, 1.75 (0.78–7.78) [n = 38]; albumin leakage, 0.58% (0.28–1.17) [n = 35]. Sputum colonization with recognised respiratory pathogens was seen in 18 of the 38 subjects but complete data on exacerbations throughout the study period was not available. CT evidence of mild, limited, tubular bronchiectasis was visualised in seven of the 38 subjects. Visual evidence of emphysema was present in 34 subjects and this included 6 of the 19 subjects who were classed as 'at risk' by the GOLD staging system.

### Disease progression

The mean interval between baseline and follow-up assessment for the whole group was 3.99 years (s.d.- 0.7). Thirty patients were smoking at baseline but 15 quit their smoking habit during the course of the study. In addition, some treatment changes were recorded over the 4 year period: inhaled corticosteroid therapy had been commenced in 8 patients and the number of patients treated with inhaled long-acting beta agonists had increased from 6 at baseline to 19.

Statistically significant disease progression over the course of the study was demonstrated by a decline in FEV_1_, FVC and gas transfer (Table 2), and reduced lung density (Table 3). Disease progression was also evident in the reassignment of patients to more advanced disease stages as defined in the GOLD classification (Figure 2). Out of the 19 patients originally classified as stage 0 at baseline, 6 had evidence of emphysema and, of these, 3 required reclassification into the 'mild' category and 2 required reclassification into the 'moderate' category at follow-up. The decline of FEV_1 _in the 19 subjects who were classified at baseline as stage 0, was 46.5 ml (95% confidence interval, 25.0 to 68.1 ml), compared to 31.5 ml (95% confidence interval, 11.8 to 51.3 ml) in the remaining subjects in GOLD stages 1 to 4. Fourteen subjects remained in the 'at risk' group after 4 years, 3 had progressed to GOLD stage 1, and 2 subjects had progressed to GOLD stage 2. No significant change was demonstrated in total lung capacity (TLC), residual volume (RV) or RV/TLC (Table 2).

**Table 2:** Change in physiological measurements over the study period (n = 56)

	Baseline (sd)	Follow up (sd)	Annual change (95% confidence interval) *p *value
FEV_1 _(L)	1.89 (0.71)	1.74 (0.68)	-37.7 ml (-52.0 to -23.4), *p *< 0.001
FEV_1 _(% predicted)	76.8 (25.1)	74.0 (27.3)	-0.8 (-1.5 to 0.0), *p *< 0.05
FVC (L)	3.38 (0.88)	3.26 (0.85)	-28.9 ml (-50.4 to -7.3), *p *< 0.01
FVC (% predicted)	109.0 (19.3)	111.0 (25.1)	0.36 (-0.5 to 1.2), Not significant
FEV_1_/FVC (%)	56.6 (0.17)	53.6 (0.17)	0.75 (0.40 to 1.1), p < 0.001
TLCO (mmol/min/kpa)	6.5 (2.4)	5.8 (2.4)	-0.15 (-0.21 to -0.09), *p *< 0.001
TLCO (% predicted)	81.8 (27.4)	76.2 (27.3)	-1.3 (-2.1 to -0.6), *p *< 0.001
KCO (mmol/min/kpa/L)	1.3 (0.4)	1.2 (0.4)	-0.02 (-0.03 to -0.01), *p *< 0.001
KCO (% predicted)	90.9 (31.6)	85.0 (31.4)	-1.4 (-2.1 to -0.7), *p *< 0.001

No change in static lung volume measurements was observed over the study period.

sd = standard deviation.

**Table 3:** Changes in CT lung density (n = 56)

	Scan 1	Scan 2	Median change/yr	Z statistic
Upper zone	-916.45	-926.7	-1.11	-3.59 (*p *≤ 0.001)
Perc15 (HU)	(-952.3 to -902.2)	(-960.1 to -912.9)	(-4.05 to -0.12)	
Upper zone	4.58	7.20	0.29	-4.05 (*p *≤ 0.001)
VI -950HU (%)	(2.31 to 15.98)	(3.74 to 19.32)	(0.02 to 0.90)	

Lower zone	-919.23	-923.24	-0.82	-2.20 (*p *< 0.05)
Perc15 (HU)	(-947.0 to -895.6)	(-948.4 to -906.1)	(-3.05 to 1.37)	
Lower zone	4.79	6.46	0.20	-3.43 (*p *≤ 0.001)
VI -950HU (%)	(1.89 to 11.35)	(3.25 to 14.62)	(-0.12 to 0.73)	

The data was not normally distributed and therefore is presented as median with interquartile range in parentheses. The Z-statistic derived from the Wilcoxon signed rank test is shown for the change in all CT parameters with *p *values in parentheses. VI = voxel index, Perc15 = 15^th ^percentile point, HU = Hounsfield Units.

The changes in physiological and CT indices observed in the 38 subjects with data on baseline inflammation were comparable to those seen in the whole group (data not shown).

### Relationship between baseline characteristics and disease progression

The loss of lung density measured from CT imaging was greater in women than men, particularly in the upper zones (Table 4). The rate of progression of CT indices in the upper zones was greater in continuing smokers than in ex-smokers (median increase in upper zone VI = 0.62% [IQR 0.14–1.57] v 0.17% [IQR -0.24–0.51] respectively, p = 0.002).

**Table 4:** Sex differences in CT density changes (n = 56)

	Men (n = 29)	Women (n = 27)	p value
Upper Zone	-0.6	-2.2	*p *= 0.006
Perc15 (HU)	(-2.11 to 0.8)	(-5.2 to -0.7)	
Upper Zone	0.17	0.46	*p *< 0.03
VI -950HU (%)	(-0.27 to 0.70)	(0.15 to 1.08)	
Lower Zone	0.23	-1.89	*p *< 0.05
Perc15 (HU)	(-1.68 to 1.71)	(-5.67 to 0.03)	
Lower Zone	0.12	0.24	not significant
VI -950HU (%)	(-0.17 to 0.57)	(0.04 to 0.79)	

The data was not normally distributed and therefore is presented as median with interquartile range in parentheses. All values are presented as a standardized annual rate of change.

VI = voxel index, Perc15 = 15^th ^percentile point, HU = Hounsfield Units

Sputum bacterial colonization at baseline did not relate to the subsequent rate of physiological and radiological progression. In addition, there was no demonstrable relationship between FEV_1 _at baseline, smoking status at baseline, sex, age, drug therapy (including inhaled corticosteroid) and the presence of visible emphysema or bronchiectasis on CT, and the subsequent rate of decline in FEV_1_.

**Figure 3:**
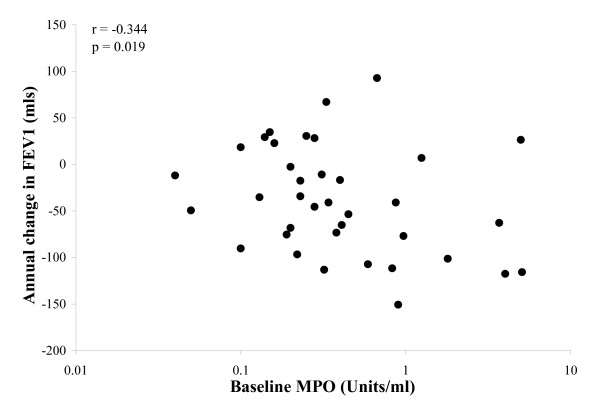
**Individual data indicating the relationship between baseline sputum myeloperoxidase activity and annual change in FEV_1_**. Spearman's correlation coefficient (r) and its significance (p) are indicated.

In the sub-group of patients with data on sputum inflammation, baseline levels of sputum myeloperoxidase correlated with the subsequent decline in FEV_1 _(rs = -0.344, p = 0.019, n = 37) (Figure 3) and FVC (rs = -0.334, p = 0.022, n = 37). This relationship was consistent for the decline in FVC (rs = -0.387, p = 0.017) following exclusion of the 7 patients with mild tubular bronchiectasis, but there was only a trend towards significance for FEV_1 _(rs = -0.254, p = 0.096).

**Figure 4:**
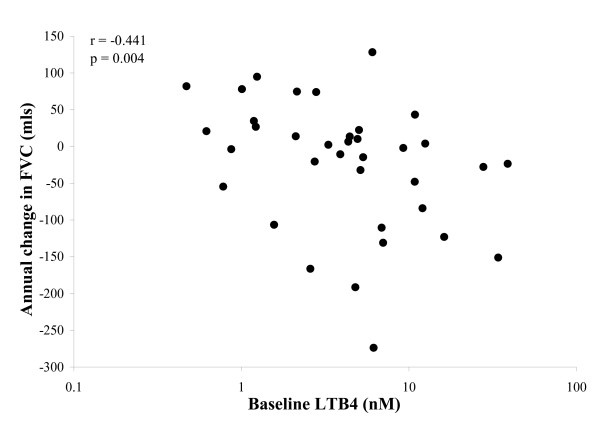
**Individual data indicating the relationship between baseline sputum LTB4 concentration and annual change in FVC**. Spearman's correlation coefficient (r) and its significance (p) are indicated.

The concentration of LTB4 at baseline correlated with the annual decline in FVC (rs = -0.441, p = 0.004, n = 36) (Figure 4) and this relationship persisted following exclusion of the 7 subjects with bronchiectasis (rs = -0.440, p = 0.008).). The LTB4 concentration was also related to the decline in TLCO (rs = -0.310; p = 0.033, n = 35) but did not correlate with the decline in FEV_1_. Albumin leak was related to decline in FVC (rs = -0.321, p = 0.03, n = 35) and TLCO (rs = -0.401; p = 0.008, n = 35). When subjects with bronchiectasis were excluded, a statistically significant relationship with TLCO could be demonstrated for LTB4 (rs = -0.432, p = 0.032, n = 29) but there was only a trend towards significance for albumin leak (rs = -0.241, p = 0.096, n = 29). Sputum IL-8 did not relate to FEV_1 _decline and the decline in KCO did not relate to any marker of progression.

**Figure 5:**
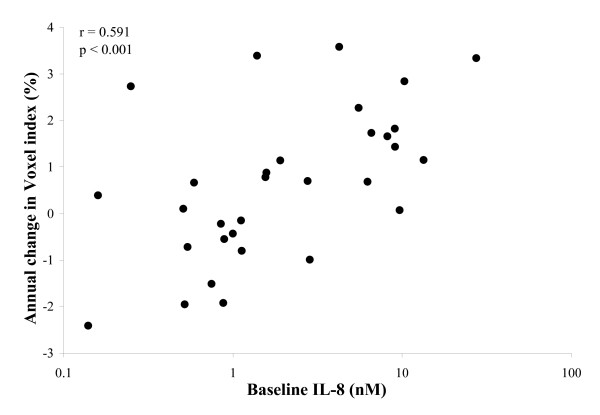
**Individual data (excluding subjects with mild bronchiectasis) indicating the relationship between baseline sputum IL-8 concentration and emphysema progression in the upper lung region (voxel index at a threshold -950HU)**. Spearman's correlation coefficient (r) and its significance (p) are indicated.

The level of IL-8 in baseline sputum correlated with the changes in CT densitometry (Table 5). These relationships remained statistically significant following the exclusion of subjects with bronchiectasis (Figure 5) and also following the exclusion of subjects who were colonised with pathogenic bacteria (data not shown).

**Table 5:** Correlation (Spearman's rho) between CT densitometry and baseline IL-8

	All patients (n = 38)	Excluding tubular bronchiectasis (n = 31)
UZ Perc15	rs = -0.464 (*p *= 0.005)	rs = -0.558 (*p *≤ 0.001)
UZ VI -950HU	rs = 0.447 (*p *= 0.005)	rs = 0.591 (*p *≤ 0.001)
		
LZ Perc15	rs = -0.447 (*p *= 0.005)	rs = -0.454 (*p *= 0.005)
LZ VI -950HU	rs = 0.353 (*p *≤ 0.02)	rs = 0.401 (*p *≤ 0.02)

UZ = upper zone, LZ = lower zone, VI = voxel index, Perc15 = 15^th ^percentile point, HU = Hounsfield Units.

## Conclusion

The current study provides unique data on the progression of physiological and radiological indices of disease severity over a four-year interval in a cohort of subjects with COPD and chronic bronchitis. In addition, it has demonstrated that markers of inflammation measured from a single sputum sample obtained at baseline are related to subsequent disease progression.

Subject selection was restricted to individuals with a clinical phenotype of chronic bronchitis but evidence of disease progression was demonstrated by changes in both physiology (Tables 2) and CT densitometry (Tables 3, 4 and 5). Progressive airflow obstruction was demonstrated over a 4 year interval with a measured rate of decline in FEV_1 _that is consistent with previous findings [25,26]. A decline in gas transfer (Table 2) and reduction in CT densitometry (Table 3) indicate that a proportion of this decline was likely secondary to progression of emphysema.

Disease progression was also evident in the re-assignment of subjects into more severe GOLD sub-groups after an interval of just four years. The progression of some individuals from GOLD stage 0 ('at risk') into the 'mild' and 'moderate' categories is pertinent because the inclusion of the stage 0 grouping in the revised GOLD guidelines has been controversial [27]. Furthermore, it is noteworthy that, despite the absence of physiological impairment, emphysema was visible on the baseline scans in 6 of the 19 'at risk' subjects. These findings justify the inclusion of stage 0 in the severity staging of COPD but suggest that use of the term 'at risk' may be falsely reassuring. Future studies are needed to further characterise this group of subjects in order to identify individuals who are at higher risk, and to enable early intervention.

The data relating sputum inflammation to subsequent disease progression in a sub-group of individuals uniquely provides direct evidence of causation. Previous studies have related airway and sputum inflammation to disease severity [3-6,28], but such data cannot distinguish cause from effect. The current study has demonstrated that higher levels of various sputum inflammatory markers are associated with a greater rate of subsequent decline in lung function and, in addition, that the level of sputum IL-8 is associated with subsequent progression of emphysema.

The latter relationship is of particular interest because sputum IL-8 is a reflection of inflammation within the proximal rather than the distal airways where emphysematous destruction occurs. Nevertheless, the association between IL-8 levels in BAL fluid and the presence of early sub-clinical emphysema [29] supports the role of IL-8 in the development of emphysema and the findings of the current study therefore suggest that inflammation in the proximal airways may be representative of pathological changes in the alveolar region. Furthermore, they imply the occurrence of a more widespread epithelial response that is consistent with the demonstration of greater IL-8 expression in the alveolar epithelial cells of smokers than of non-smokers [30].

These findings are of importance in the consolidation of current understanding of the pathogenesis of COPD and suggest a potential method for identifying individuals at greater risk of disease progression. Nevertheless, it is recognised that there are some limitations to the data. It was not possible, using single measurements, to consistently demonstrate the presence of a relationship between inflammation and indicators of disease progression for all measures. However, single data points may not correlate merely because of intra-patient variability, and the use of repeated measures would likely have improved the consistency of the associations between inflammation and markers of disease progression.

It is also recognised that the exclusion of subjects with bronchiectasis reduced the number of identifiable associations between inflammation and disease progression. The decision that assessment should be performed using data for the whole group and also following the exclusion of subjects with radiological evidence of bronchiectasis was made *a priori *because of the recognised influence of bronchiectasis on bronchial inflammation [31]. Notwithstanding the reduction that this exclusion had on the size of the study population, and the likelihood of identifying a statistically significant relationship, such a relationship was nevertheless maintained for some parameters. Consequently, it cannot be concluded that the relationship between sputum inflammation and disease progression is merely a reflection of the presence of bronchiectasis. Furthermore, it is important to recognise, given the clinical phenotype of 'chronic bronchitis', that the presence of mild tubular bronchiectasis on CT was likely a co-incidental finding of uncertain clinical significance.

There are a number of other confounding variables that merit discussion. Smoking status changed in 15 subjects and it is probable that subsequent inflammation and disease progression would have been influenced by these changes. Nevertheless, it is unsurprising that we were not able to demonstrate the influence of changes in smoking status on disease progression in such a small sample size. The adjustment of COPD-related therapy in accordance with normal clinical practice was reported in 24 individuals (8 patients were commenced on inhaled corticosteroids and the number of patients prescribed long-acting beta agonists increased from 6 at baseline to 19 at follow-up). We did not explore the potential influence of these therapeutic changes on the progression of disease because it is generally understood that current pharmaceutical treatment does not modify the natural history of COPD and the current study was not powered for such analyses.

The inflammatory response that is the pathological hallmark of COPD is a complex process involving several different mechanisms, although the relative importance of these pathological processes remains undetermined. The current study of subjects with COPD and chronic bronchitis demonstrates that prospective measurements of markers that relate to neutrophilic inflammation in sputum are associated with the rate of subsequent disease progression. Consequently, the data are of critical importance in establishing the role of this inflammatory mechanism in the pathogenesis of COPD. In addition, they indicate that markers of lung inflammation in COPD are of potential value for risk stratification in clinical practice and, furthermore, may assist in guiding contemporary strategies for the development of effective disease modifying therapy.

## Competing interests

Dr Parr's and Dr White's salaries were paid for by a non-commercial grant from Bayer plc and Dr Parr acts as a consultant for Talecris Biopharmaceuticals and Roche. Professor Stockley has lectured widely for non-promotional purposes to several pharmaceutical companies (Glaxo Smith Kline, Bayer and Eli Lilly) and acts on advisory boards with an interest in COPD (Astra Zeneca, Glaxo Smith Kline, Bayer Biologicals, Schering-Plough and Baxter Pharmaceuticals) and as a consultant (Etiologics). In addition, significant non-commercial research grants have been awarded by Astra Zeneca and Bayer.

## Authors' contributions

Every author has contributed to reviewing the paper. DGP performed the image analysis and drafted the manuscript. AJW performed the statistical analysis and contributed to drafting the manuscript. DB performed the laboratory work. PJG reported the visual appearance of the CT images. RAS is the principal investigator of the project, obtained funding of and supervised the project. All authors read and approved the final manuscript.
